# Dichotic turncoats: Lateralization of auditory processing in two dichotic listening tasks using melodies and syllables

**DOI:** 10.1371/journal.pone.0333510

**Published:** 2025-09-26

**Authors:** Simon Knobloch, Philipp Haul, Saskia Rusche, Heiko Paland, Darius Zokai, Moritz Haaf, Jonas Rauh, Christoph Mulert, Gregor Leicht

**Affiliations:** 1 Department of Psychiatry and Psychotherapy, Psychiatric Neuroimaging Branch, University Medical Center Hamburg-Eppendorf, Hamburg, Germany; 2 Department of Child and Adolescent Psychiatry, Psychosomatics and Psychotherapy, Ludwig-Maximilians-University of Munich, Munich, Germany; 3 Centre of Psychiatry, Justus Liebig University Giessen, Giessen, Germany; Gabriele d'Annunzio University of Chieti and Pescara: Universita degli Studi Gabriele d'Annunzio Chieti Pescara, ITALY

## Abstract

When confronted with dichotically presented syllables, right-handed healthy individuals tend to consciously perceive syllables presented to the right ear more often. This phenomenon, known as the right-ear advantage, is driven by delayed processing of information from the left ear in left temporal auditory cortex due to its indirect relay through the corpus callosum. In contrast, less is known about about the corresponding mechanisms for stimuli processed in the right temporal hemisphere. In this study, we developed a melody-based dichotic listening paradigm designed to induce a left-ear advantage. This novel paradigm, alongside a classical syllable-based paradigm was tested in 40 healthy right-handed participants. We also examined the influence of musical education on lateralization of auditory processing. Our results revealed a significant left-ear advantage for the perception of dichotically presented melodies and replicated established findings of a right-ear advantage for syllables. No group differences emerged between participants with or without current or past musical practice. However, among those with musical training, a greater number of years of practice was associated with a reduced right-ear advantage for syllables and an increased report of melodies presented to the left-ear. These findings suggest that the left-ear advantage in dichotic perception of melodies reflects right hemispheric processing of musical stimuli. Moreover, monitoring of the left ear seems to be altered by musical practice. Future research using neuroimaging techniques will be necessary to confirm this finding.

## Introduction

**Fig 1:**
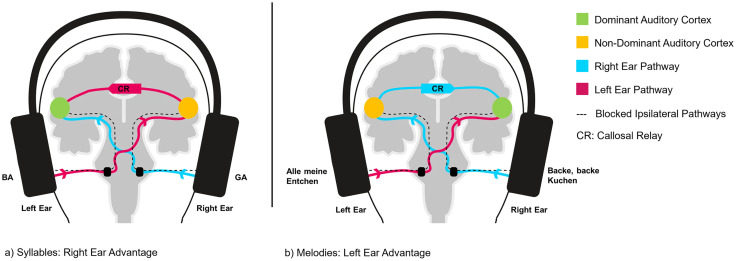
The Callosal Relay Model. Neural pathways in dichotic listening. a) depicts the neural basis assumed to drive the REA for syllables. Syllables presented to the right ear immediately reach the auditory cortex of the left hemisphere. Syllables presented to the left ear have to be relayed across the corpus callosum, arriving in the auditory cortex of the left hemisphere with a delay. b) depicts the assumed mirrored mechanism of the LEA for melodies. Melodies presented to the left ear immediately reach the auditory cortex of the right hemisphere. Melodies presented to the right ear have to be relayed across the corpus callosum, arriving in the auditory cortex of the right hemisphere with a delay.

The right-ear advantage [REA] in healthy right-handed populations in dichotic listening [DL] paradigms is a frequently observed behavioral phenomenon of lateralized cortical auditory processing [1–4]. DL paradigms utilize headphones to confront participants with two different concurrent auditory stimuli, challenging them to discriminate the perceived auditory input [e.g. 5]. Typically, participants presented with competing speech related auditory input (e.g., syllables containing a consonant-vowel structure like ‘ba’, ‘ga’, ‘da’ with different stimuli presented to the right and left, e.g. left: ‘ba’, right: ‘da’) perceive a larger share of stimuli from the right ear consciously [3,4,6]. Early on, the origin of side-based ear advantages in responses to stimuli has been interpreted to be the result of a lateralized processing in specialized cortical areas of auditory stimuli in a dominant hemisphere [4]. With ipsilateral auditory pathways assumed blocked through the dichotic presentation [7], left ear stimuli would have to be relayed via the corpus callosum to be processed and perceived consciously. This transcallosal delay was assumed to drive the REA [8]. Fig 1a) depicts the neural pathways for stimuli presented to both ears respectively.

In support of this theory, lesions [7] and low structural integrity of the splenial and posterior regions of the corpus callosum are associated with an increase of the REA, hinting at the interhemispheric auditory pathway passing through this region of the corpus callosum. Assessment of the functional and effective connectivity in the gamma band further supports the mechanistic notion of transcallosal relay. In accordance with the theoretical assumptions, left ear reports are associated with an increased connectivity between the primary and secondary auditory cortices directed from the right hemisphere to the left hemisphere [9]. This connectivity can be modulated through transient alternating current stimulation in the gamma-band range (center frequency 40 Hz), leading to an increase or decrease of the REA depending on the intrinsic phase asymmetries [10]. A subsequent study revealed a decrease of lateralization, i.e. an increased connectivity, when endogenous and exogenous phase lags matched closest as compared to sham and farthest stimulation conditions [11]. Transcranial direct current stimulation on the other hand failed to alter the REA for syllable recognition [12]. In forced attention versions of the dichotic listening paradigm, evidence for a reciprocal inhibition of contralateral secondary auditory cortices was found. This contrariwise connectivity is understood to prevent multiple stimuli from being consciously perceivable [13]. In patients with schizophrenia and in a pharmacological model of this disease, a reduced or missing REA is related to the occurrence of auditory verbal hallucinations [AVH], a finding which has been confirmed in a meta-analysis. [14–18]. Interestingly, AVH-patients also show a diminished ear preference when imagining unilateral voices [19], while healthy right handed participants typically prefer the right ear when imagining a voice unilaterally [20]. Structurally, an increased posterior callosal connectivity in AVH patients compared to schizophrenia patients without AVH supports a link between dichotic listening phenomenon and symptom [21]. Moreover, EEG studies have shown that the reduced REA related to AVH in schizophrenia is accompanied by a significantly higher functional connectivity in the gamma frequency range between bilateral auditory cortices, when syllables presented to the left ear are perceived [14]. Consequently, AVH and altered speech perception in schizophrenia have been proposed to originate from interhemispheric miscommunication, highlighting the clinical significance of DL research [22].

It is important to consider that our capacity to encode and integrate auditory input is not confined to speech-related information but extends to other modalities of auditory processing. It stands to reason that a left-ear advantage [LEA], resulting from right hemispheric dominance can be found as well. Accordingly, DL paradigms using different types of non-verbal stimuli have been applied. However, evidence for a LEA is less well consolidated than the REA: Research reports LEAs for musical stimuli [23–25], for musical stimuli only in female participants [26], for the identification of emotional content of musical stimuli [27] and language stimuli [28]. A study investigating the dichotic processing of stimulus length found a REA for both language and musical stimuli [29]. One discussed reason for the conflicting results is the heterogeneity of musical stimuli as compared to simple syllables. Musical stimuli surpass most syllables in length, complicating dichotic presentation and may lead to a LEA only at increased speed [24]. Further, participants’ processing of musical stimuli may differ in accordance with their musical education or practice, which has a higher variability than participants’ education in language understanding. Accordingly, musicians were shown to have no LEA or an REA in response to melody stimuli [24]. This difference between musicians and non-musicians in the processing of melodies has been discussed to be an effect of additional analytic resources located in the left hemisphere which musicians acquire due to their musical training.

The heterogeneity of results on lateralization of musical processing highlights the need for further research. Additionally, evidence on whether the REA and LEA are similarly found in one sample is relatively scarce. Only one previous study from [23] combined syllables and musical instrument stimuli, finding both a LEA for musical instrument stimuli and a REA for syllables. This study aims to replicate both, i.e. a REA for syllables and a LEA for melody stimuli, in a healthy, right-handed sample. We use melody snippets from well-known German children’s songs and a well-tested syllables paradigm. We hypothesize that melodies will be reported predominantly from the left ear, while syllables will be predominantly reported from the right ear. In view of the previous findings by [24] we hypothesize that musical education diminishes the LEA effect. As we assume equality in mechanism driving the effects, we hypothesize that the grade of lateralization for syllables and melodies is linked in individuals.

## Methods

### Participants and procedure

40 healthy right-handed participants were recruited through blackboard postings at the University Medical Center Hamburg. All participants were fluent in German, thus able to understand the task instructions and familiar with the German children’s songs. Inclusion criteria were normal or corrected-to-normal vision and right-handedness as determined by the Edinburgh Handedness Inventory (sufficient right-handedness for study participation was determined as a Laterality Quotient ≥ 30) [30]. Exclusion criteria included a history of previous and current psychiatric disorders, current substance abuse, past or present neurological disease, abnormal hearing, and left handedness.

Participants completed the Edinburgh Handedness Inventory, a sociodemographic questionnaire, and a questionnaire about musical education and practice. To ensure normal hearing, all participants underwent a tone threshold audiometry, with abnormal hearing defined as an auditory threshold > 20 dB and an interaural difference > 15 dB. The experimental task consisted of two dichotic listening conditions. After finishing the task, participants filled out a questionnaire assessing recognizability and familiarity of the stimuli.

The study was conducted in accordance with the latest version of the Declaration of Helsinki and approved by the ethics committee of the Hamburg medical chamber. No financial compensation was provided for participation. All participants provided written informed consent before participating in the study. The data for this study was collected between 16.06.2023–27.02.2024.

### Dichotic listening task

**Fig 2:**
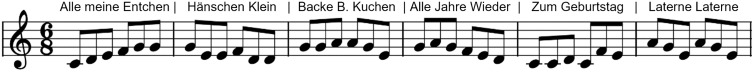
Melodies. Score of the melody-snippets used in the experiment. Note that each bar corresponds to one stimulus. The bars were played individually and not in succession.

The DL task consisted of two conditions. In one condition 6 Syllables (‘ba’, ‘ga’, ‘da’, ‘pa’, ‘ka’, ‘ta’) were presented dichotically (e.g., simultaneous presentation of ‘ba’ to the left and ‘da’ to the right ear). The dichotic listening task with syllables has been widely used internationally [e.g. 23,31,32] as well as in our research lab [5,14,33]. In the other condition, 6 melody-snippets were presented dichotically. Following Messerli and colleagues, [24] well-known German children’s songs (an English translation of the title is given in brackets after each title) were chosen for this task: Alle meine Entchen (All my ducklings), Backe backe Kuchen (Bake, bake a cake), Hänschen klein (Little Hans), Zum Geburtstag viel Glück (Happy birthday to you), Alle Jahre Wieder (Year after year) and Laterne Laterne (Lantern, lantern). From those melodies short snippets were created. The snippets consisted of 6 notes each. All snippets were harmonized into the same pitch range (c’-a’), rhythm and articulation (eighth note, non-legato) and key (C major). See Fig 2 for the scores of the used melody snippets. As Messerli and colleagues reported a LEA solely at an increased tempo [24], the snippets were presented with 320 bpm. museScore (MuseScore Ltd, Limassol, Cyprus) was used for the creation of the .wav-audiofiles and the visualization of the melodies’ score. After each stimulus presentation participants were asked to choose one melody or syllable, they thought to have perceived from the full choice of melodies resp. syllables with a computer mouse (right click: choose, left click: confirm choice). Participants were instructed orally and in written form to listen to the stimuli and choose the one they thought they heard best. Before the experiment participants trained the recognition of the syllables and melodies under binaural (i.e., the presentation of the same stimulus on both ears) listening conditions. For the melodies the training consisted of two parts in which participants were trained to recognize at first the original melodies at normal speed and then the melody-snippets from the experiment at the experimental speed. Each experimental condition consisted of 120 trials (30 possible pairings, each pairing administered 4 times) divided into 6 blocks. Melody- and syllable-blocks were delivered in alternating order. Participants were randomly assigned to either start with a melody- or syllable-block. Presentation software (neurobs.com, RRID:SCR_002521) was used to deliver the stimuli and record the participants’ answers. The melody snippets can be downloaded upon request to the corresponding author.

### Analysis

The count of trials in which participants reported to have perceived the stimulus presented to the right ear and left ear respectively were calculated. Further, the count of stimuli logged without presentation on either side was calculated (error-rate). To account for participants with incomplete data, a relative value for both right ear reports [RER] and left ear reports [LER] was generated by division of the counts by the unilateral number of appearances of the stimulus (for single stimuli analyses) or by the number of trials (for analyses concerning all CV-stimuli or melody-stimuli). Outliers were defined as participants outside an interval from Q1 – 1.5 x interquartile range to Q3 + 1.5 x interquartile range and were excluded from further analysis. In those cases, selective attention processes were inferred to explain the lateralization. From this data the lateralization index [LI] was calculated as: (RER – LER)/ (RER + LER) x 100. Note that positive values indicate a right-ear preference and negative values indicate a left-ear preference. From this data a 2x2 repeated measurement ANCOVA with side (LER/RER) and stimulus-type (syllables/ melodies) as factors was calculated as the primary outcome measure. Age and self-reported sex were included as covariates. Using post-hoc paired t-tests, the LIs were compared across the two task-conditions. Also, each LI was individually tested against zero (LI > 0 for syllables and < o for melodies) in one-sided one-sample t-tests. Further, to test the amount of variance explained through individual stimuli, single-stimuli LIs were tested against zero in equal one-sided one-sample t-tests. The melody snippets recognizability and familiarity ratings’ association with lateralization was assessed through Pearson’s correlation.

In a subsequent analysis the influence of musical education on the lateralization of melody recognition was assessed. Using independent-sample t-tests LIs were compared between participants that played instruments currently or in their lifetime and those who did not. Among those who had played instruments, the association between years of musical activity and the LIs as well as LER and RER individually was assessed through Pearson’s correlation.

To assess whether the high error-rate in melody-recognition influenced the lateralization of musical perception, a median split analysis was conducted as an independent-sample t-test comparing the LI in two groups of 17 participants each. An analysis for outliers (interval from Q1 – 1.5 x interquartile range to Q3 + 1.5 x interquartile range) in melody error-rate was conducted to assess whether participants should be excluded from main analyses.

All statistical analysis were conducted using JASP 0.19.3.0 (RRID:SCR_015823). Matlab 2023a (Mathworks Inc., Natick, Massachusetts, USA, RRID:SCR_001622) was used to read-out logfiles and count the participants’ answers. The threshold for statistical significance was set to p < .05. Multiple comparisons were accounted for through Bonferroni-Holms correction.

## Results

### Demographics

Of the 40 recruited healthy participants, two subjectively right-handed participants were excluded as the Edinburgh Handedness Inventory revealed mixed- or left-handedness (Edinburgh Handedness Inventory Laterality Quotient of −10 and −60 respectively). Further, one participant who selected the same melody stimulus throughout the entire experiment and three outliers (see Methods 2.4) were excluded, leaving a final sample of 34 participants. Questionnaires assessing socioeconomic, motivational and musical activity were completed by 33 participants. Characteristics of the study sample are presented in Table 1, while data on musical activity is summarized in Table 2.

**Table 1:** Sample characteristics.

Characteristics	Descriptives
Age (mean ± SD)	25.53 ± 8.0
Self-reported sex	
Male, n	24
Female, n	10
Years of education (mean ± SD)	15.74 ± 2.74
EHI Laterality Quotient (mean ± SD)	80.91 ± 23.37

Table 1 legend: Sociodemographic characterization of the sample. For dimensional variables, mean and standard-deviation are reported. For nominal variables the n of each value is reported. EHI = Edinburgh Handedness Inventory.

**Table 2:** Musical Activity.

Variable	Descriptives
Years of musical activity (mean ± SD)	4.55 ± 4.16
**Instrument played current**	
Yes, n	6
No, n	27
**Instrument played lifetime**	
Yes, n	27
No, n	6
Hours of musical activity, weekly (mean ± SD)	3.15 ± 4.77

Table 2 legend: The variables describing the samples musical practice are reported. or dimensional variables, mean and standard-deviation are reported. For nominal variables the n of each value is reported.

### Main outcome

**Fig 3:**
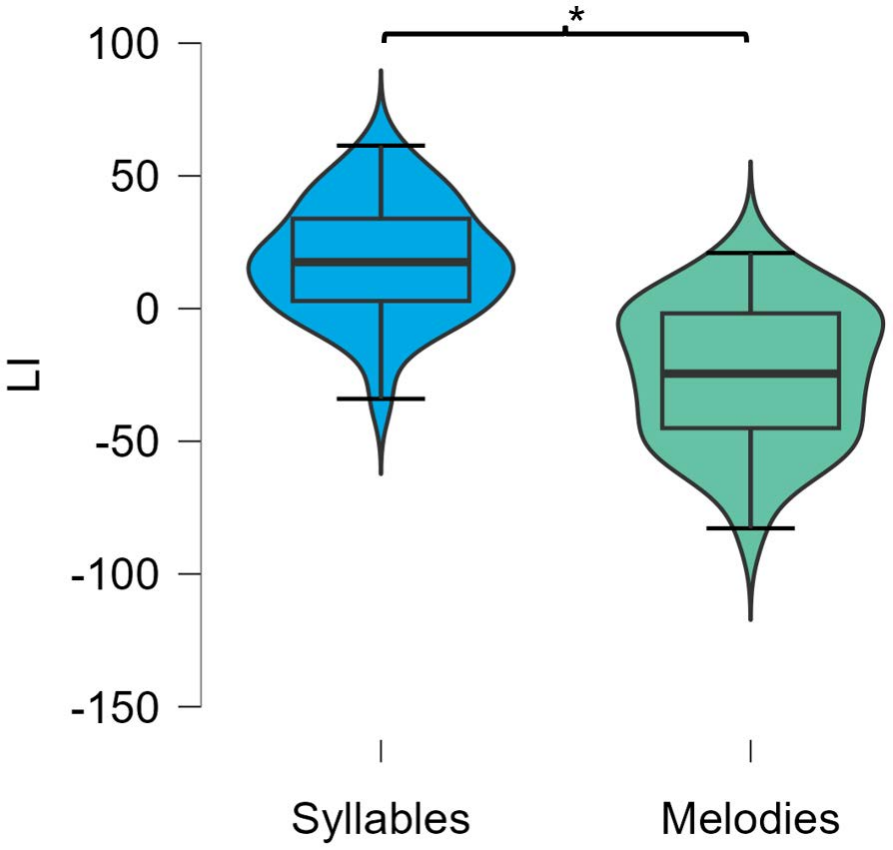
Primary Outcome Variables. Violin-plots of primary outcome variables: Left Ear Reports (LER) and Right Ear Reports (RER) for syllables and melodies.

The main repeated measurement ANCOVA revealed a significant side by stimulus type interaction (F = 13.7, p < .001, df = 1, η²p = 0.31). No significant main effects for the factors side or stimulus-type were observed. Further, no between participants effect regarding age and sex were observed as well as no interaction with within subject measures. See Table 3 for the full outcome of the main effect ANCOVA. Fig 3 shows violin-plots of the variables, that entered the ANCOVA (LER and RER for syllables and melodies).

**Table 3:** Main effect repeated measurement ANCOVA.

Cases	Sum of Squares	df	Mean Square	F	p	η²p
**Within Subjects Effects**						
Stimulus	199.77	1	199.77	1.85	0.183	0.06
Stimulus ✻ Sex	10.65	1	10.65	0.10	0.755	0.003
Stimulus ✻ Age	0.11	1	0.11	0.001	0.975	<0.001
Residuals	3341.78	31	107.80			
Side	163.00	1	163.00	0.73	0.400	0.02
Side ✻ Sex	0.21	1	0.21	<.001	0.976	<0.001
Side ✻ Age	130.08	1	130.08	0.58	0.451	0.02
Residuals	6932.75	31	223.64			
Stimulus ✻ Side	1492.76	1	1492.76	13.73	< .001*	0.31
Stimulus ✻ Side ✻ Sex	263.81	1	263.81	2.43	0.129	0.07
Stimulus ✻ Side ✻ Age	234.52	1	234.52	2.16	0.152	0.07
Residuals	3369.86	31	108.71			
**Between Subjects Effects**						
Sex	24.52	1	24.52	0.23	0.632	
Age	0.38	1	0.38	0.00	0.952	
Residuals	3241.68	31	104.57			

Table 3 legend: Results of the two-factor repeated measurement ANCOVA. Factors: Stimulus (syllables and melodies), Side (right, left). Sex and Age were included as covariables.

**Fig 4:**
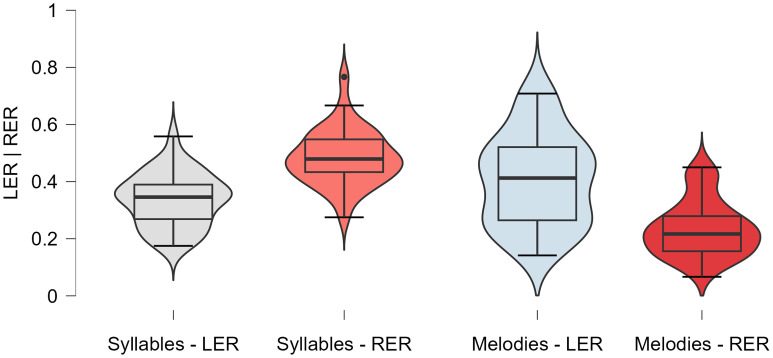
Lateralization Indices. Violin-plots of lateralization indices of melodies and syllables. The asterisk marks the significant difference.

Post-hoc paired-sample t-tests revealed a significant difference of the LIs (t = 8.45, df = 33, p < 0.001, Cohen’s d = 1.45) of Syllables (LI (mean ± SD): 18.28 ± 21.24) and melodies (LI (mean ± SD): −24.56 ± 25.85). Both LIs were shown to differ from zero through one-sample t-tests (Syllables: t = 5.02, df = 33, p < 0.001, Cohen’s d = 0.86; melodies: t = −5.54, df = 33, p < 0.001, Cohen’s d = −0.95). Note that these three t-tests were treated as independent questions and not corrected for multiple comparisons. Fig 4 depicts violin-plots of both LIs. The well-known right-ear advantage for the recognition of syllables in our study was accompanied by a similar left-ear advantage for the recognition of melodies. No correlation between the lateralization-indices was observed (Pearson’s r = .224, p = .202). See Table 4 for detailed descriptive results of the outcome measures. The melody-recognition task yielded a higher error-rate compared to the syllable-recognition task. An independent-sample t-test found no significant difference (t = −1.63, df = 32, p = 0.113, Cohen’s d = −0.56) in melody LI between participant below (LI (mean ± SD): −31.61 ± 25.95) and above (LI (mean ± SD): −17.51 ± 24.48) the median error-rate. Note that the descriptive evidence hints at lateralization increasing with error-rate. Although the range of errors between individual participants differed widely, no outliers were detected. Thus, no participants were excluded from main analyses retrospectively.

**Table 4:** Behavioral outcome measures.

Variable	Descriptives
Syllables: RER (mean ± SD)	48.46 ± 9.77
Syllables: LER (mean ± SD)	33.53 ± 9.30
Melodies: RER (mean ± SD)	23.52 ± 9.77
Melodies: LER (mean ± SD)	40.71 ± 15.94
Syllables: LI (mean ± SD)	18.28 ± 21.24
Melodies: LI (mean ± SD)	−24.56 ± 25.85
Syllables: error-rate (mean ± SD)	18.09 ± 7.07
Melodies: error-rate (mean ± SD)	35.78 ± 18.72

Table 4 legend: Descriptives statistics of outcome-variables. Means and standard-deviations are reported.

### Influence of musical activity

Independent-sample t-tests revealed no significant group differences of current or lifetime musical practice with regard to lateralization of both melodies and syllables (see Table 5).

**Table 5:** Independent sample t-tests: musical practice.

Variable 1	Descriptives	Variable 2	Descriptives	t	df	p	Cohen’s d
LI syllables: currently instrument	12.15 ± 24.51	LI syllables: currently no instrument	19.72 ± 21.12	0.77	31	0.445	0.35
LI melodies: currently instrument	−29.54 ± 31.31	LI melodies: currently no instrument	−25.14 ± 23.96	0.39	31	0.703	0.17
LI syllables: lifeitme instrument	17.15 ± 22.25	LI syllables: lifeitme no instrument	23.71 ± 18.94	0.67	31	0.509	0.30
LI melodies: lifetime instrument	−28.23 ± 26.59	LI melodies: lifetime no instrument	−15.64 ± 12.29	1.12	31	0.270	0.51

Table 5 legend: Lateralization Indices (mean and standard-deviation) compared among persons practicing music currently or ever in independent sample t-Tests.

**Fig 5:**
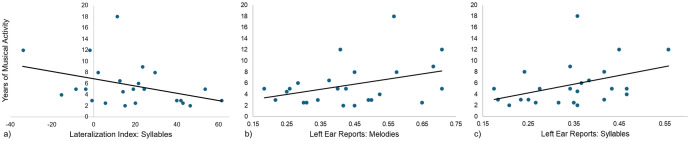
Association of Musical Activity and Behavior. Scatterplots of the association of years of musical activity and a) the lateralization index of syllables, b) left ear reports of melodies and c) left ear reports of syllables. a) -c) depict only trendwise associations, that did not survive the correction for multiple comparisons.

Years of musical activity were inversely associated with a weaker laterization of syllable recognition (see Fig 5a)) but not melody recognition (Syllables: Pearson’s r = −0.415, p = 0.039, p Holms = 0.078; melodies: Pearson’s r = −0.072, p = 0.733, p Holms = 0.733; corrected for two comparisons). This association does not survive correction for multiple comparisons. Previous research has shown musical education advances the left ear monitoring capabilities [34]. Thus, we analyzed the influence of musical education on LER and RER separately. Years of musical activity were correlated (corrected for four comparisons) with a higher percentage of LER (see Fig 5b)-5c)) in melodies and trended significant correlation in syllables (Syllables: Pearson’s r = 0.443, p = 0.026, p Holms = 0.104; melodies: Pearson’s r = .426, p = .034, p Holms = 0.102) but not RER (Syllables: Pearson’s r = −0.329, p = .129, p Holms = 0.129; melodies: Pearson’s r = .369, p = .069, p Holms = 0.138). Again, these associations did not survive the correction for multiple (four) comparisons.

No correlation was observed between the weekly hours of musical activity and the LIs, LER or RER. Note that only six participants currently practiced music, reducing the sample size for this analysis severely.

### Analysis of single stimuli

One-sample t-tests of the single stimulus LI and their respective lateralization values reveal that all syllables and melodies lead to lateralization to the right resp. left ear. Table 6 lists the details of the single-stimulus analyses. For both stimulus-types, p was Bonferroni-Holms-corrected for a total of six comparisons respectively.

**Table 6:** Single stimulus analysis.

Stimulus	LI (mean ± SD)	T	df	p	p Holms	Cohen’s d
ba	41.35 ± 50.90	4.60	31	0.00003	0.00015	0.200
ga	38.382 ± 35.29	6.34	33	0.0000002	0.0000012	0.222
ta	21.02 ± 44.01	2.66	30	0.006	0.006	0.199
da	16.66 ± 31.27	3.06	32	0.002	0.004	0.199
ka	13.71 ± 18.01	4.44	33	0.00005	0.0001	0.200
pa	16.08 ± 28.28	3.32	33	0.001	0.003	0.190
Alle meine Entchen	−20.57 ± 39.37	−3.05	33	0.002	0.008	0.188
Hänschen klein	−27.94 ± 30.00	−5.43	33	0.000003	0.000015	0.211
Backe backe kuchen	−18.26 ± 45.66	−2.30	32	0.014	0.028	0.188
Alle Jahre Wieder	−15.38 ± 44.55	−2.01	33	0.026	0.026	0.188
Zum Geburtstag viel Glück	−22.65 ± 47.22	−2.80	33	0.004	0.012	0.180
Laterne Laterne	−38.07 ± 39.01	−5.61	32	0.000002	0.000012	0.21

Table 6 legend: Lateralization Indices (mean and standard-deviation) compared in one-sample t-Tests. Syllables are tested > 0, melodies are tested < 0. p is corrected for 6 comparisons respectively using Bonferroni-Holms method.

## Discussion

In our study, we investigated perceptual lateralization in dichotic listening tasks involving syllable and melody stimuli to reexamine the well-established REA for language-related auditory processing and explore the lateralization of music-related auditory processing. Building on more than 60 years of research, the main finding demonstrates a significant REA for syllables, reflecting left-hemispheric specialization for linguistic stimuli [3,5,7,23,33]. In line with our hypothesis, our melody snippets elicited a robust LEA for melody recognition, suggesting a right-hemispheric dominance for music-related processing. Our findings corroborate previous reports on reversed lateralization of musical stimuli [23–25,35]. This finding hints at musical stimuli from the right ear to be disadvantaged in conscious processing. A relay through the corpus callosum from the left to the right hemisphere is a possible mechanism.

In our sample, we found no group-differences among musicians and non-musicians regarding the LI of melodies. This conflicts with data from previous research [24,35,36]. Note that in two studies, musicians were compared to non-musicians [35,36], while in our study non-professional musicians were ranked according to the quantity of free-time musical practice. Yet, also free-time musicians have been shown to differ from participants without musical experience in the processing of melodies [24]. Possibly the heterogeneity of group sizes in our sample (27 vs. 6 life-time musical practice, 6 vs. 27 current musical practice) disguised behavior differences. Interestingly, we found a trendwise association of years of musical activity with a weaker lateralization of syllable stimuli. This surprising finding can be interpreted in the light of previous findings of an increased recruitment of right-hemisphere areas in musicians during speech-perception and reports of superior left-ear monitoring skills in adults practicing music [34]. Previous research has described an influence of musical activity on the percentage of melodies reported from the right ear but not the left ear, i.e. musicians reporting a higher percentage of right ear stimuli [24,35,36]. In our sample, a trendwise correlation between the years of musical activity emerged only for the percentage of LER. This surprising finding supports previous evidence of musical activity on left-ear, i.e. right hemisphere, processes [34,37]. Although group-wise statistics and associations might not be directly comparable, our findings hint at a more mixed picture of the influence of musical activity on the lateralization auditory processing in the brain. It has been argued, that for musicians, speech and music processing are less differentiable than in non-musicians due to a language-similar approach to music perception [35]. From our findings neural alterations in music-practicing adults can only be inferred, yet, our data tends to support the view that right hemispheric processes are influenced by musical activity rather than left hemispheric processes. Alternatively, the increase in LER can be interpreted from the perspective of altered interhemispheric connectivity. A stronger transcallosal connectivity between auditory cortices might increase LER if assuming left-hemispheric processing of melodies in musicians. Musical practice can be interpreted, thus, as strengthening the callosal relay in order to enable musicians to use both ears to perceive and process music in the left-hemisphere. Conclusions from these findings must be drawn with caution though, as none of the associations survived the correction for multiple comparisons. A repetition of the paradigm with a larger sample would be needed to analyze these seemingly subtle differences.

Future research will have to analyze the neural activity during melodies reported from the left and right ear. Robust evidence supports the view, that a delay caused by a callosal relay drives the REA found for syllables. Information presented to the weak ear (i.e., information, that primarily arrives in the non-dominant hemisphere) reaches specialized left-temporal areas with a relay-associated delay [5]. Utilizing lagged phase synchronization, a significantly increased functional connectivity in the gamma band was observed, only when stimuli presented to the left ear were reported by participants. Furthermore, a stronger functional connection between the auditory cortices was associated with a decrease of the REA. This finding has been understood to result from stronger functional connectivity enabling more information from the left ear to reach the left hemisphere through the corpus callosum. This notion is further supported by evidence from effective connectivity analysis [33]. Syllable reports from the left ear have been found to be associated with a directional connectivity from the right- to the left auditory cortex. No contrariwise connectivity has been found for either RER or LER of syllables. This confirmed the postulated importance of a transcallosal transfer delay for stimuli arriving in the right (see Fig 1a)), i.e. contralateral to the language-dominant left hemisphere. Our DL-paradigm, yielding both a robust REA and LEA, enables future combined analysis of neural substrates of lateralized perception of speech and music (for a proposed mechanism see Fig 1b)) related stimuli in both musicians and non-musicians. An interesting perspective on these mirrored phenomena has been described in dichotic listening paradigms conducted with whistled variants of languages. A study comparing lexically identical DL paradigms of vocal and whistled Turkish found a diminished right ear advantage for whistled Turkish as compared to vocal Turkish [38]. Future research might further disentangle the contributions of both hemispheres in the analysis of complex sounds by using our double-paradigm in patients with aphasia [39] or amusia [40].

In our sample, we found a somewhat smaller size of right-ear lateralization (18.28 vs. circa 23–24) and an increased error-rate (18.09 vs. circa 10–11) for Syllables, compared to previous administrations of the experiment in our lab [5,14,33]. Our experimental setup included interchanging blocks of melodies and syllables, which might have created interference effects that added to the grade of attentional demand the task posed. This might have induced cognitive fatigue explaining the increased error-rate and the deviation from previously reported lateralization indices. Although no significant influences of self-reported sex and age have been found in our sample, the lower degree of lateralization for syllables may well be explained through the very young sample of this study, as the REA for speech stimuli is known to steadily grow with age [41]. Previous analyses of a large sample tested on a syllables paradigm report a significant sex by age interaction on the REA, with younger adults having a more pronounced REA in males [41]. Our young adult sample does not support this, although the structure of our sample might have hindered the uncovering of these subtle effects. In our sample, we found neither a general influence of sex, nor a sex by stimulus interaction. This adds to the body of research reporting no sex influence on LEA as compared to studies reporting a LEA only in female participants [26]. Significant differences over the hormonal cycle in female participants for the LEA have been reported, which might explain the conflicting results on sex-influence.

A previous study from the nineties reports that only some of the stimuli, when analyzed separately, contribute to the phenomenon of lateralized perception. Of the mostly used syllables (see Methods 2.3) it was found that ‘ta’ but not ‘da’ leads to a REA [42]. Note that this study used a target-syllable detection approach, while our study gave participants the choice of all syllables after each trial. To our knowledge, no comparable analysis exists for melody stimuli. In our study, we did not replicate the findings of syllables without a REA and found a LEA for all melodies used. The analysis of lateralization of single melodies and syllables showed small differences in the degree of lateralization though. Differences in the grade of lateralization hint at a subtle effect on processing depending on the characteristics of the stimuli used. According to our findings the lateralization of auditory processes seems to be a strong effect, that surpasses this influence of single-stimuli characteristics. We interpret this finding as supportive of a general opposite lateralization of speech and music processing in the brain.

### Limitations

This study aimed to develop and implement a novel DL-paradigm that robustly yields a REA and LEA to enable the analysis of lateralized auditory processing. To that end, the paradigm was tested on 40 participants. The sample size and the unequal sex-distribution prevented a detailed analysis of previously reported age and self-reported sex influences on the lateralized processing of syllables and melodies. Furthermore, the acquired sample differed vastly in musical practice and did not include professional musicians, which limits the validity of the analyzed influence of musical practice on the lateralization of music processing. DL research has traditionally recruited right-handed participants. We followed this approach in our study, which limits the generalizability of our results to the general population. The importance of assessing left-handed individuals in neuroscience has been stated before [43] and should encourage further studies of language and music processing in a larger and mixed sample. Although yielding a robust LEA independent of the participants error-rate, the novel paradigm showed a generally high and highly variant error-rate for musical stimuli among participants. Thus, future research might try to create improved melody paradigms, that are more directly comparable in difficulty to the traditional syllable paradigm. The implementation of the novel paradigm was conducted collecting only behavioral data, so any purported underlying neural mechanisms for LEA were only inferred by transfer of previous evidence for the REA.

### Conclusion and outlook

In a sample of healthy adults, we observed a REA for syllables and a LEA for melodies in a dichotic listening task. Musical education had a comparable effect on left-ear performance for both melodies and syllables, while the specific 6 stimuli used in each task had no major influence. We propose that mirrored neural mechanisms lead to the REA for syllables and the LEA for melodies. Future research using neuroimaging techniques will unravel how brain activation and connectivity shape the lateralized auditory processing of music and speech stimuli.

## Supporting information

S1 File Raw experimental data. This spreadsheet includes the raw experimental data. Participants’ age at the date of assessment and gender (1 = female, 0 = male) are included. The musical practice is described in two binary variables: ever_played_instrument and currently_playing_instrument (1 = yes, 0 = no) and the variables year_of_musical_education and hours_of_weekly_musical_practice. Furthermore, the laterality quotient of the Edinburgh Handedness Inventory is included. The variables describing the experimental data include the following abbreviations: LI = lateralization index, RER = right ear report, LER = left ear report, AE = Alle meine Entchen, HK = Hänschen klein, BK = Backe backe Kuchen, JW = Alle Jahre wieder, ZG = Zum Geburtstag viel Glück, LL = Laterne laterne. Variable names are given in the following format: Index(LI, LER, RER, errorate)_stimulus(syllables, melodies, single syllables [ba, ga, da, ka, ta, pa], single melodies [AE, HK, BK, JW, ZG, LL]).
(CSV)
